# The Effect of Forced Melt Flow by a Rotating Magnetic Field and Solid/Liquid Front Velocity on the Size and Morphology of Primary Si in a Hypereutectic Al-18 wt.% Si Alloy

**DOI:** 10.3390/ma18112581

**Published:** 2025-05-31

**Authors:** Dimah Zakaraia, András Roósz, Arnold Rónaföldi, Zsolt Veres

**Affiliations:** 1 Institute of Physical Metallurgy, Metal Forming & Nanotechnology, University of Miskolc, 3515 Miskolc, Hungary; zakaraia.dimah@uni-miskolc.hu (D.Z.); zsolt.veres@uni-miskolc.hu (Z.V.); 2 HUN-REN TKI, Materials Science Research Group, University of Miskolc, 3515 Miskolc, Hungary; rarnold@digikabel.hu

**Keywords:** hypereutectic Al-Si alloys, RMF, primary Si, solid/liquid front velocity

## Abstract

Hypereutectic Al-Si alloys containing primary Si exhibit unique material properties that make them suitable for various industrial applications. Understanding the characteristics of primary Si is crucial for predicting the effect of solidification conditions on the microstructure of these alloys. This paper presents a comprehensive characterisation study of primary Si in hypereutectic alloys. This study provides a detailed analysis of the size, distribution, and morphology of primary Si, providing valuable insights into the alloy structure, mechanical properties, and even the performance of the production process. The effect of forced melt flow by a rotating magnetic field (RMF) and solid/liquid front velocity on the size and morphology of primary Si in a hypereutectic Al-18 wt.% Si alloy was investigated. The purpose of using the RMF technique during the solidification process of Al-Si alloys is to enhance the alloy’s microstructure by inducing electromagnetic stirring. The hypereutectic samples were solidified at five different front velocities (0.02, 0.04, 0.08, 0.2, and 0.4 mm/s), under an average temperature gradient (G) of 8 K/mm, in a crystalliser equipped with an RMF inductor. Each sample was divided into two parts: the first solidified without stirring, while the second underwent electromagnetic stirring using RMF at an induction (B) of 7.2 mT. The results revealed that increasing the front velocity during solidification refined the primary Si in stirred and non-stirred parts. In non-stirred parts, it decreased dendritic forms and increased star-like Si, while polyhedral shapes remained nearly constant. Stirred parts showed stable Si morphology across velocities. Higher velocities also promoted equiaxed over elongated Si forms in both parts.

## 1. Introduction

In materials science and engineering, the solidification behaviour of metallic alloys holds significant importance, due to its profound impact on the mechanical, thermal, and structural properties of the resultant materials. Among the numerous alloy systems, Al-Si alloys have garnered extensive attention for their remarkable combination of being lightweight, having corrosion resistance, and having excellent castability [1]. However, hypereutectic Al-Si alloys, characterised by a Si content surpassing the eutectic composition, require careful microstructure control to optimise performance in corrosion-critical applications [2].

Generally, the Al-Si eutectic is treated as an alloy exhibiting an irregular structure. However, this irregularity typically comprises regions with regular eutectic morphology and areas with complex, branched formations [3]. More detailed analysis of irregular eutectic growth distinguishes both: a eutectic structure resulting from its formation under a stationary state (regular structure growth), branching morphology connected with the extremally perturbed solid/liquid interface (marginal stability state) of the non-faceted eutectic phase, and many areas showing intermediate morphologies. It can be concluded that irregular eutectic growth oscillates between stationary and marginal stability states [4].

Theoretical models of solidification provide essential insights into the mechanisms that govern phase formation and microstructure development. For instance, Saifutdinov and Lebedev [5] modelled hydrogen segregation during solidification, showing how pressure gradients and solute diffusion affect microporosity formation. Additionally, the model developed by Pequet et al. [6] provides valuable insights into the formation of microporosity and macroporosity in Al-Si alloys by accounting for interdendritic flow and pressure drops within the mushy zone during solidification.

The solidification of hypereutectic Al-Si alloys involves the early nucleation of primary Si before the eutectic reaction occurs. The size, distribution, and morphology of primary Si particles significantly influence material properties such as wear resistance, thermal expansion, and strength [7]. Therefore, characterisation and evaluation of the primary Si in hypereutectic Al-Si alloys are of great interest and importance [8,9]. Understanding the factors influencing primary Si formation and solidification behaviour is essential for tailoring Al-Si alloys to diverse engineering applications.

Among the key parameters affecting solidification microstructure are the solid/liquid front velocity ($v_{SL}$) and forced melt flow, such as inducing the melt using a rotating magnetic field (RMF). The s/l front velocity can influence heat transfer rates and solute distribution. Similarly, applying an RMF during solidification has demonstrated the ability to modify melt flow patterns, nucleation kinetics, and grain refinement [10,11].

Several studies have investigated the effect of different solidification parameters on the primary Si of hypereutectic alloys. For example, researchers Xu and Jiang described the effect of cooling rate and melt overheating temperature on the hypereutectic structure by observing the morphologies and size changes of the primary Si particles under different solidification conditions. With an increase in the temperature of melt overheating, they found that the morphologies of primary Si changed from a star-like to an octahedral shape, and the size of the particles decreased. As the cooling rate increases, the size of the primary Si becomes smaller [12]. They found that a reasonable inference could be drawn that a larger Si–Si cluster size would likely promote the inception of star-like primary Si nuclei, while a smaller Si–Si cluster size would favour the origin of octahedral primary Si nuclei.

Ullah and colleagues [13] examined the morphologies of primary Si crystals during the solidification of Al-Si alloys containing 17–38 wt.% Si. Their findings revealed that lower Si concentrations led to Si morphologies resembling fishbones or star-like shapes. However, as the Si content increased, the growth pattern shifted to larger plates that tended to grow in layers.

Yan and co-authors [14] investigated the influence of the Sr-modifier and the serpentine pouring channel process on primary Si in their study. Their analysis involved quantifying the size of primary Si particles by determining their equivalent diameter. The findings demonstrated that in the A390 alloy, employing the water-cooled copper serpentine pouring channel and Sr-modifier in combination resulted in significant refinement of primary Si particles, reducing their size to 25.2–28.5 µm.

Despite the significant progress in understanding the solidification behaviour of primary Si in hypereutectic alloys, a comprehensive investigation into the characteristics of primary Si in hypereutectic Al-Si alloys, and the effects of front velocity and the application of an RMF during solidification on the primary Si, is still lacking. This paper aims to characterise the primary Si particles and investigate the effect of front velocity and forced melt flow by applying an RMF during solidification to the primary Si in a hypereutectic Al-Si alloy. The size and morphology of the primary Si were analysed using new measurement methods.

## 2. Materials and Methods

### 2.1. Materials and Experiments

**Figure 1:**
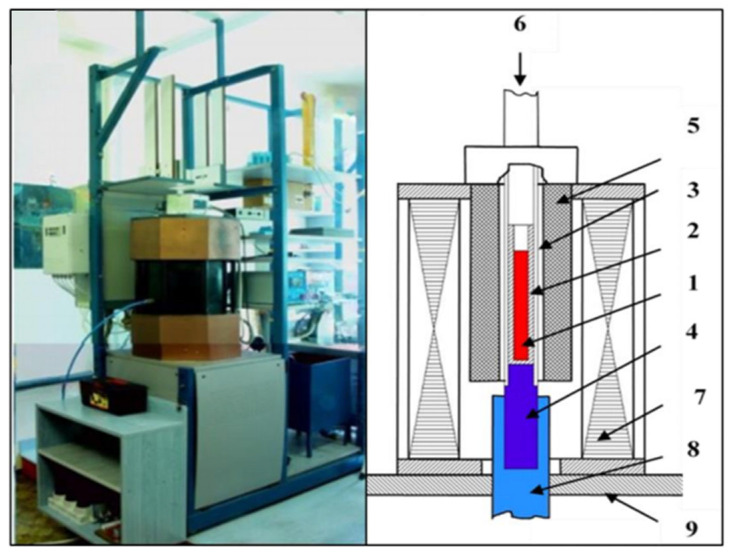
A schematic overview of the solidification facility, showing the following components: (1) sample, (2) alumina capsule, (3) quartz tube, (4) copper cooling core, (5) furnace with four heating zones, (6) step motor, (7) RMF inductor, (8) water-cooling system, and (9) basement [16,17].

The initial material used for the experiments was an Al-18 wt.% Si alloy in the form of ingots, which were remelted before solidification. Hypereutectic Al-18 wt.% Si alloy samples were prepared using a controlled unidirectional solidification process—Al with a purity of 99.95 wt.% and Si with a purity of 99.95 wt.%. The unidirectional solidification process was carried out using the vertical upward Bridgman method. The samples, cylindrical in shape, measured 8.5 mm in diameter and 90 mm in length. The solidification process was conducted in a crystalliser equipped with a high rotating magnetic field (HRMF) [15,16,17]. The crystalliser comprised a furnace with a programmable temperature controller, a water-cooling unit, a stepper motor to regulate sample movement, and 13 type K thermocouples positioned at varying lengths around the sample. Additionally, a MagnetoHydroDynamic (MHD) inductor generated an RMF during solidification (Figure 1). The unidirectional solidification was achieved by vertically translating the sample relative to the furnace chamber (The equipment and all of its parts are developed by our research group and the developer is Arnold Rónaföldi, Miskolc, Hungary, EU).

The experimental setup involved solidifying five hypereutectic samples using different sample movement velocities. The s/l front velocities were 0.02 mm/s, 0.04 mm/s, 0.08 mm/s, 0.20 mm/s, and 0.40 mm/s. The average temperature gradient (G) value was 8 K/mm. The initial half of each sample was solidified without applying magnetic stirring. The second half of each sample was solidified under the influence of an RMF with an intensity of B = 7.2 mT (Table 1). The material composition and all solidification parameters, including the temperature gradient and RMF intensity, were kept constant. The only varied parameter was the front velocity. The s/l front velocity $\left(v_{SL}\right)$ and temperature gradient (G) were calculated from the “Temperature-Time” function developing along the length of the samples during the solidification process. The distance from the bottom of the sample was determined based on the known positions of thermocouples placed along the 90 mm length. The solid/liquid front velocity was calculated using the time at which each thermocouple recorded the temperature dropping below the liquidus temperature (638 °C, determined using Differential Scanning Calorimetry (DSC)). Using the formula v = Δx/Δt, where Δx is the spacing between thermocouples and Δt is the time difference of the s/l front passage, the velocity profile was obtained along the sample.

### 2.2. Microstructural and Macrostructural Analysis

The samples were prepared through standard metallographic procedures to analyse the primary Si particles accurately. This involved sectioning, grinding, polishing, and etching using a 0.5 *V*/*V*% hydrofluoric acid (HF) solution for 15 s [18]. An optical microscope was employed to observe the macrostructure and microstructure of the alloy samples. High-resolution images were acquired, capturing the distribution and morphology of the primary Si. Micrographs were taken at 100× magnification to capture primary Si phases.

### 2.3. Image Analysis

**Figure 2:**
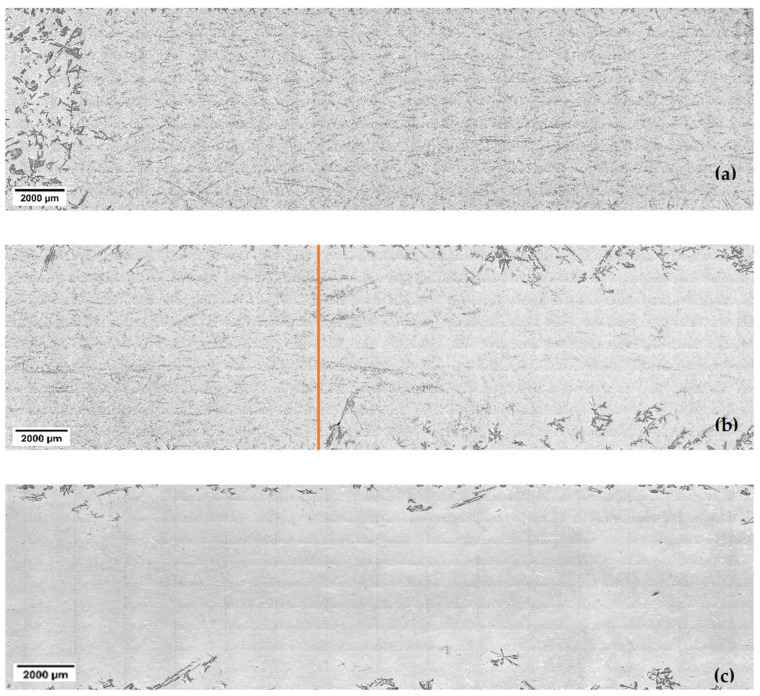
The macrostructure of the Al-18 wt.% Si hypereutectic sample observed at a front velocity of 0.4 mm/s, showing (**a**) the non-stirred part, (**b**) the transition zone, and (**c**) the stirred part. The orange line indicates where the stirring begins.

Each sample was cut into three pieces: the beginning of the non-stirred part (Figure 2a), the transition zone between non-stirred and stirred parts (Figure 2b), and the sequel of the stirred part (Figure 2c). While only one sample is shown in Figure 2, trends were consistent across all samples. ImageJ 1.52a, a free, open-source image analysis software developed by the National Institutes of Health (NIH), LOCI, USA. was employed to process the acquired micrographs. This involved segmenting the particles from the background matrix using a thresholding algorithm. The software distinguished between primary Si particles and the surrounding aluminium matrix. Following this, primary Si particles were analysed, and particle size measurements were extracted, including the area and perimeter. Shape factors, such as circularity and roundness, were also computed to assess the morphology of the primary Si particles.

### 2.4. Quantitative Measures

#### 2.4.1. Size Factor

This study evaluated the size of primary Si as an equivalent diameter. The equivalent diameter represents the diameter of a hypothetical circle with the same area as the particle’s actual shape. In the context of primary Si particles in hypereutectic Al-Si alloys, the equivalent diameter provides a convenient way to quantify and compare particle sizes, enabling adequate material characterisation and property prediction. The equivalent diameter (*D_eq_*) is calculated using the following equation [19]:

(1) $$D_{eq}=2\sqrt{\frac{A}{\pi }}$$

where *A* is the area of a primary Si particle.

#### 2.4.2. Shape Factors

This section introduces two crucial quantitative measures for characterising the morphology of primary Si particles in hypereutectic alloys: circularity and roundness. These dimensionless measures provide valuable insights into the shape characteristics of the particles, which are fundamental to understanding the microstructure and properties of these materials. These shape factors are defined as follows:

##### Circularity

Circularity is a shape factor that evaluates how close a particle is to a perfect circle, considering its overall roundness and the smoothness or irregularities of its perimeter (P). Particles with jagged or irregular boundaries will have lower circularity values, indicating greater deviation from a smooth, circular form. The circularity (C) of each primary Si particle is calculated using the following equation [20]:

(2) C=4π×(A) (P^2)

where *P* is the perimeter of a primary Si particle.

The circularity value ranges from 0 to 1 (perfect circles). As the value approaches 0.0, it indicates an increasingly elongated and irregular shape.

##### Roundness

Roundness (R) measures how closely an object’s shape resembles a perfect circle, focusing on its overall form, rather than edge details. Mathematically, it can be expressed as follows [20]:

(3) R=4×[A] (π×[Major axis]^2)

where the “*Major axis*” refers to the length of the object’s major axis. Roundness is related to crystal geometry, and its value ranges from 0 (elongated particle) to 1 (perfect circle). As the value approaches 0.0, it indicates an increasingly elongated shape.

## 3. Results and Discussion

### 3.1. Qualitative Analysis of the Solidified Samples

**Figure 3:**
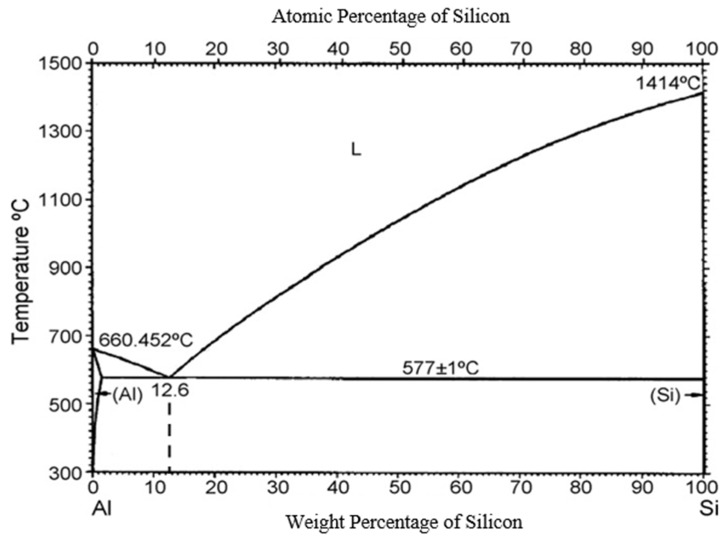
Phase diagram of Al-Si alloys.

The Al-Si binary alloy is a eutectic system, and its eutectic composition and temperature were determined to be 12.6 wt% Si and 577 °C, respectively (Figure 3). The solidification of the hypereutectic Al-Si alloy starts with primary Si and finishes with Al-Si eutectic, and the solidified samples contain primary Si and Al-Si eutectic. The volume percentage of primary silicon in the Al-18 wt% Si alloy is approximately 6.98%. The solidification path situated on the liquidus line is usually completed by the eutectic and begins just at the nominal solute concentration. The solidification path can appear many times (be repeated), as is clearly proved in the case of the growth of a single crystal decorated with some eutectic stripes (layers) [21].

**Figure 4:**
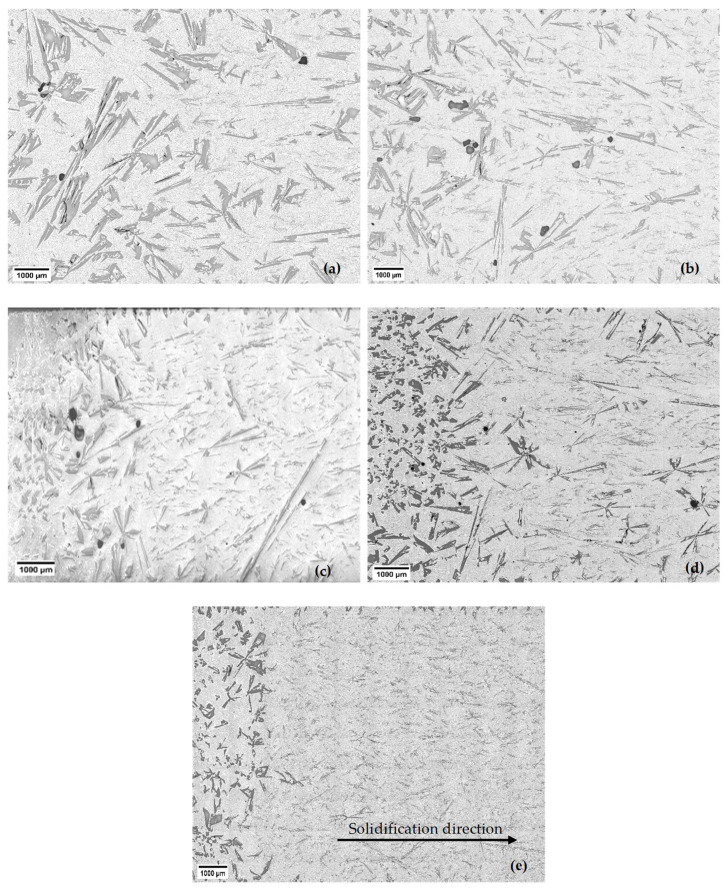
The macrostructure of the non-stirred parts of the Al-18 wt.% Si hypereutectic samples at different front velocities: (**a**) 0.02 mm/s, (**b**) 0.04 mm/s, (**c**) 0.08 mm/s, (**d**) 0.20 mm/s, and (**e**) 0.40 mm/s.

**Figure 5:**
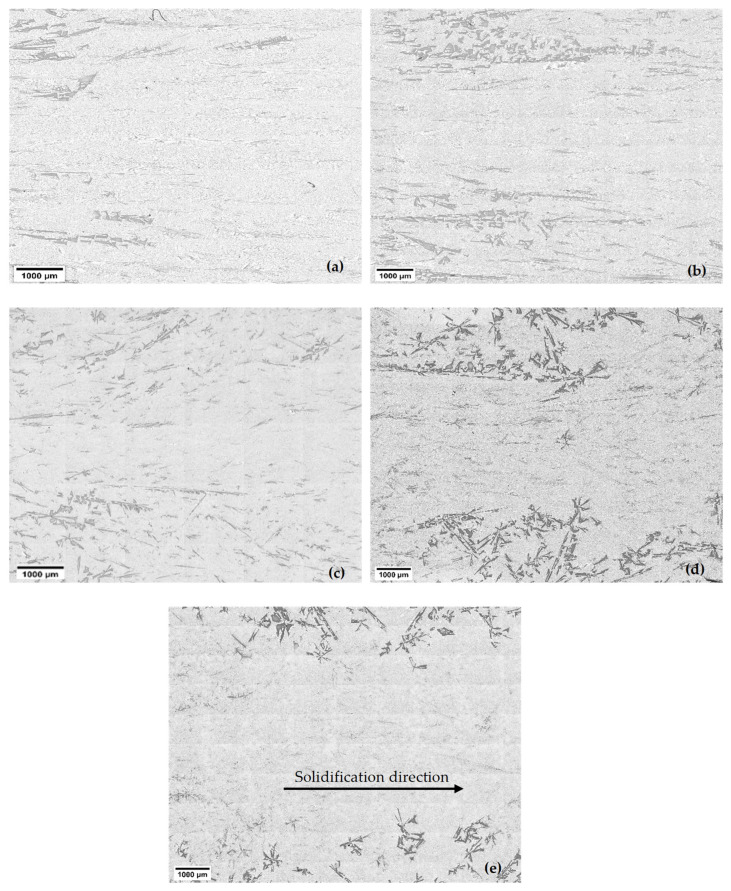
The macrostructure of the stirred parts of the Al-18 wt.% Si hypereutectic samples at different front velocities: (**a**) 0.02 mm/s, (**b**) 0.04 mm/s, (**c**) 0.08 mm/s, (**d**) 0.20 mm/s, and (**e**) 0.40 mm/s.

Examining the solidified samples under a microscope provided valuable insights into the distribution of primary Si particles within the eutectic matrix (Figure 4 and Figure 5). Figure 4 and Figure 5 are part of the micrographs seen in Figure 2 for all velocities. The analysis revealed an intriguing phenomenon known as macrosegregation in the non-stirred and stirred parts. At the beginning of each sample, where the first part of solidification was carried out, more primary Si was present than in the later solidified parts. It is visible that the higher the sample movement velocity, the less volume there is, and the smaller the size of the primary Si.

**Figure 6:**
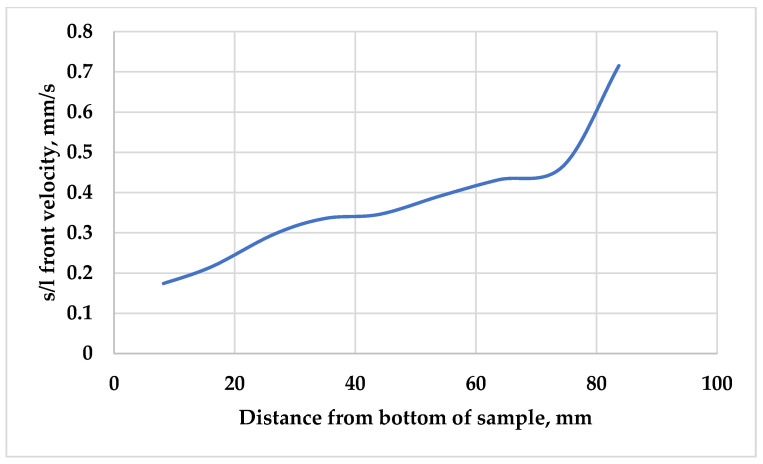
The solid/liquid front velocity as a function of distance from the bottom of the sample, with an s/l front velocity of 0.4 mm/s.

Figure 6 presents the s/l front velocity as a function of position along the sample with an average set velocity of 0.4 mm/s. At the beginning of the solidification, the $v_{SL}$ was very low due to the nature of the experiment. Due to temperature gradients and dynamic heat transfer during solidification, the local velocity naturally varied and increased near the top of the sample, briefly reaching values around 0.7 mm/s. While only one sample is shown in Figure 6, the trends were consistent across all samples. During solidification, a soft zone was built up in which the concentration of Si at the s/l front was almost 18%, and it decreased toward the bottom of the soft zone, where the eutectic was solidifying. Because of this concentration gradient, Si diffusion occurred from the upper part of the sample to the bottom of the sample. The transferred Si atoms at the lower and colder part of the mushy zone were solidified at the existing solid Si phases. Because of this phenomenon, the concentration gradient in the soft zone was almost constant over time, and the Si particles grew until the eutectic front enriched them.

With lower sample movement velocities, and so with a lower $v_{SL}$, the diffusion had more time to perform the above-described macrosegregation, so more and bigger Si particles were observed at the bottom of the samples solidified with a lower $v_{SL}$.

When the magnetic stirring was switched on (at almost half the length of the samples), the macrostructure of the samples suddenly changed. Most of the primary Si appeared concentrated at the edges of the samples, compared with the middle part, which contained mainly eutectic (Figure 3). The RMF induced two distinct types of melt flow during solidification: azimuthal and axial flows [22,23]. Azimuthal flow is perpendicular to the axis of the solidification direction, while axial flow runs parallel to it. These flows redistribute the primary Si particles from the central axis toward the borders of the sample [16]. As a result of the combination of the azimuthal and axial flows, the motion becomes spiral. The structure of the stirred parts is likely a result of the rotating spiral flow, which moves the primary solid parts to the edges, where they can swim up and melt, or become stuck and grow. The stuck parts can be seen in the microstructure.

**Figure 7:**
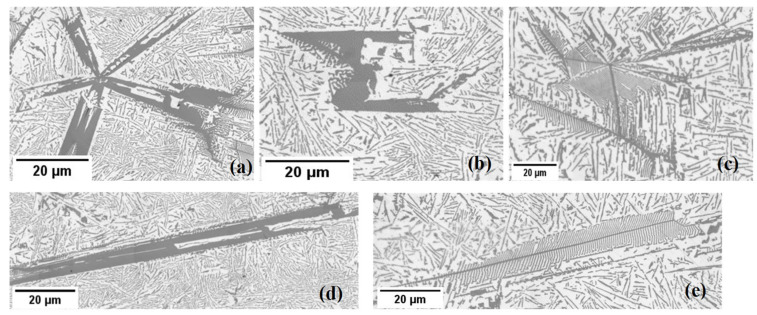
The morphologies of primary Si in the studied hypereutectic alloy: (**a**) a star-like structure exhibits arms originating from a large nucleus and growing along their axes; (**b**) an equiaxed polyhedral structure with multiple flat facets; (**c**) an equiaxed dendritic structure with tree-like branching; (**d**) an elongated polyhedral (coarse plate-like) structure; (**e**) an elongated dendritic (feather-like) structure with fine, feather-shaped branches. Microstructures were obtained from both stirred and non-stirred parts of the samples.

Figure 4 and Figure 5 show the samples’ stirred and non-stirred sections, revealing primary and eutectic Si particles with various shapes and sizes. Eutectic Si particles are depicted as plate-like structures, while primary Si particles exhibit diverse morphologies, including dendritic, polyhedral, and star-like configurations (Figure 7).

- Star-like: This structure exhibits needle arms, suggesting growth along their axes (planes) originating from a large nucleus (Figure 5a).
- Polyhedral: Polyhedral structures have multiple flat faces or facets, indicating the presence of well-defined crystal planes during growth. This morphology appears under two subtypes: equiaxed polyhedral morphology (Figure 7b) and elongated polyhedral, or, as adopted in the literature, coarse plate-like morphology (Figure 7d) [24].
- Dendritic: Dendritic structures are characterised by branching, tree-like patterns. The dendritic morphology appears under two subtypes: an equiaxed dendritic shape (Figure 7c) and an elongated dendritic shape (feathery) (Figure 7e). Feather-like configurations imply delicate, elongated structures resembling feathers.

Despite the qualitative observations, accurately describing differences in the morphology, size, and distribution of primary Si in stirred and non-stirred parts remains challenging, due to its irregular shape. Image analysis techniques would be beneficial for more precisely characterising these differences.

### 3.2. Quantitative Analysis of the Solidified Samples

#### 3.2.1. Size of Primary Si Particles

**Figure 8:**
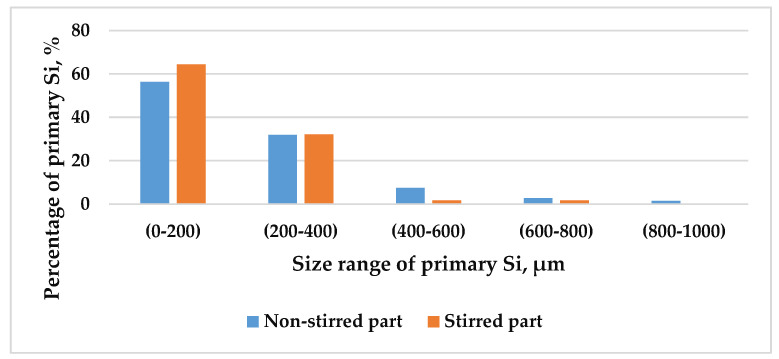
The percentage of primary Si as a function of its size range in the stirred and non-stirred parts of the sample that solidified at $v_{SL}$ = 0.02 mm/s.

ImageJ analysis software was used to quantify the size and distribution of the primary Si in the microstructure. The equivalent diameter of each primary Si particle was used to measure the size of primary Si. The measurements were analysed by classifying the primary Si equivalent diameters into different ranges, ranging from 0–200 to 800–1000 µm. This procedure was followed in the stirred and non-stirred parts of five samples. Figure 8 shows the percentages of the primary Si in the stirred and non-stirred parts of the sample that solidified at a front velocity of 0.02 mm/s. While only one sample is shown, the trends are consistent across all samples. The results show that at 0.02 mm/s velocity, the primary Si percentage decreased with increasing size in both the stirred and non-stirred regions. The same trend was observed at higher velocities.

**Figure 9:**
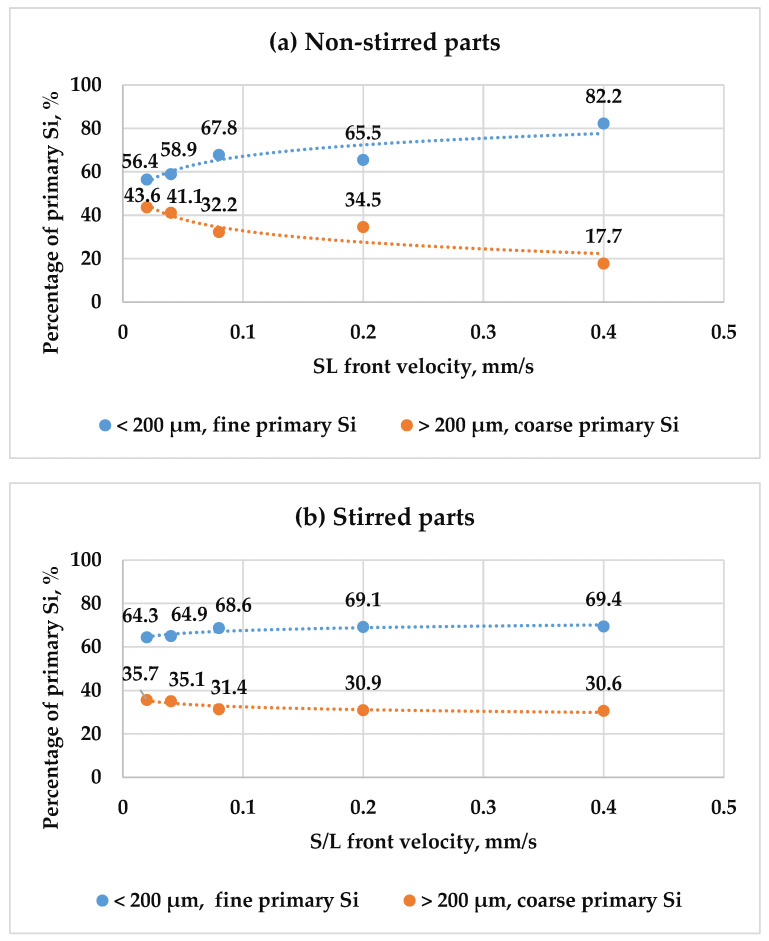
The percentages of fine and coarse primary Si particles as a function of front velocity in both the (**a**) non-stirred and (**b**) stirred parts of the five samples.

For deeper insight, the primary Si particles were classified into two categories: fine primary Si, measuring less than 200 µm, and coarse primary Si, exceeding 200 µm in size. This classification was applied to both stirred and non-stirred parts across all samples. Figure 9 illustrates the distribution of primary Si relative to front velocity, showcasing a notable trend: as velocity increases, the percentage of fine primary Si rises, while that of coarse primary Si diminishes, in both the stirred and non-stirred parts, indicating the refinement of primary Si with heightened front velocity in the stirred and non-stirred parts. This phenomenon arises from the shortened solidification time at higher velocities, facilitating a finer microstructure.

Increasing the s/l front velocity generally leads to a higher cooling rate. Typically, the growth of primary silicon occurs as silicon atoms attach to the surfaces of existing primary silicon particles. Thus, the diffusion of silicon atoms is crucial for developing primary silicon. As the cooling rate increases, it becomes more difficult for silicon atoms to diffuse, which substantially restricts the growth of primary silicon. Furthermore, a higher cooling rate leads to easier and faster nucleation. Consequently, with an increase in the cooling rate, the size of the primary silicon particles is significantly reduced.

Additionally, applying an RMF during solidification amplified the percentage of fine primary Si while reducing that of coarse primary Si compared to in the non-stirred parts. Notably, the non-stirred parts contained a greater percentage of coarse primary Si particles than the stirred parts. The combined effect of heightened front velocity and RMF during solidification synergistically enhanced primary Si refinement in the hypereutectic alloy. This is attributed to the constrained diffusion of solute atoms, resulting in increased nucleation sites for primary Si particles and, thus, a refined microstructure.

The calculated equivalent diameter provides valuable information about the size and distribution of primary Si. This information is essential for predicting material properties such as strength, hardness, wear resistance, and thermal behaviour. Larger primary Si particles can act as stress concentrators, affecting the alloy’s fracture behaviour. Smaller particles enhance solidification and subsequent microstructural refinement.

#### 3.2.2. Shape of Primary Si

The shape factors, including circularity and roundness, were assessed for ten particles of each primary Si shape, including polyhedral, plate-like, star-like, equiaxed dendritic, and elongated dendritic (feather-like) shapes, followed by the calculation of their respective averages and standard deviation (Table 2).

Numerous trends can be identified from the data of Table 2. These trends will be analysed individually based on each shape descriptor.

- **Circularity**

Circularity, expressed by Equation (2), hinges on the particle’s perimeter; increasing the perimeter reduces circularity and enhances roughness. Consequently, as a shape becomes smoother and more rounded, the circularity tends towards one, exemplified by polygon shapes with a circularity greater than 0.2, and to a lesser extent, star shapes exhibit a circularity between 0.1 and 0.2. Conversely, as a shape becomes less round or smoother, the circularity approaches zero, demonstrated by dendritic shapes, which have the highest perimeter, with a circularity below 0.1 (Table 2).

**Figure 10:**
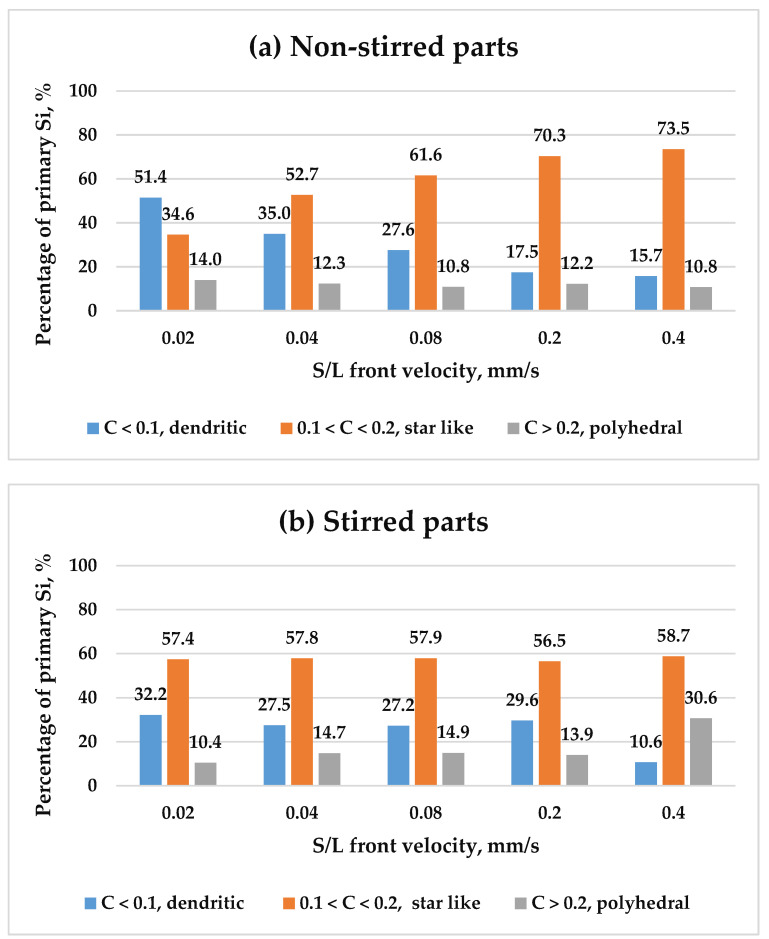
The percentages of primary Si in terms of their circularity as a function of front velocity in both the (**a**) non-stirred and (**b**) stirred parts of the five samples.

For the analysis, the primary Si particles were categorised into three groups based on their circularity: dendritic particles (circularity < 0.1), representing the roughest particles; star-like particles (0.1 < circularity < 0.2), less rough; and polyhedral particles (circularity > 0.2), the smoothest. These categories were analysed across both stirred and non-stirred sections of all samples. Figure 10 illustrates the variation in the percentages of these primary Si particle types as a function of front velocity in both stirred and non-stirred sections. Observations indicate that an increase in front velocity decreases the percentages of dendritic primary Si particles, while increasing the percentage of star-like particles, in non-stirred sections. The percentage of polyhedral particles seems almost constant. The percentages of dendritic, star-like, and polyhedral silicon shapes in the stirred parts appear relatively constant across varying front velocities. Minor fluctuations are observed, but the overall trend indicates stability in the distribution of primary Si shapes. As the front velocity increases in non-stirred parts, the cooling rate changes, affecting the way in which Si particles form. Higher velocities promote rapid solidification, which can suppress the growth of dendritic structures while favouring the formation of star-like particles. Dendritic growth requires slower cooling, which is disrupted at higher front velocities, leading to a reduction in dendritic Si.

An increase in the s/l front velocity in the stirred regions does not appear to affect the morphologies of primary silicon. The distribution of different primary silicon morphologies remains nearly unchanged. It is believed that the morphology of primary silicon in the solid state is closely linked to the nucleation process in the liquid melt. During solidification, regardless of variations in front velocity, a consistent liquid structure leads to similar primary silicon nuclei forming in the melt, resulting in minimal morphological differences. Therefore, this can indicate that different s/l front velocities in the stirred parts hardly affect the nucleation of various morphologies of primary Si from the liquid melt. In other words, increasing the front velocity in the stirred parts hardly affects the morphologies of primary Si in the solid state.

- **Roundness**

As expressed by Equation (3), roundness hinges on the particle’s maximum diameter. As an object becomes more elongated or less circular, its roundness value tends toward zero. This is exemplified by elongated polyhedral (plate-like) shapes and elongated dendritic (feathery) shapes, which exhibit roundness values < 0.1. Conversely, objects with highly rounded forms approach a roundness value of one. This is illustrated by equiaxed polyhedral, star, and equiaxed dendritic shapes with roundness values > 0.1 (Table 2).

**Figure 11:**
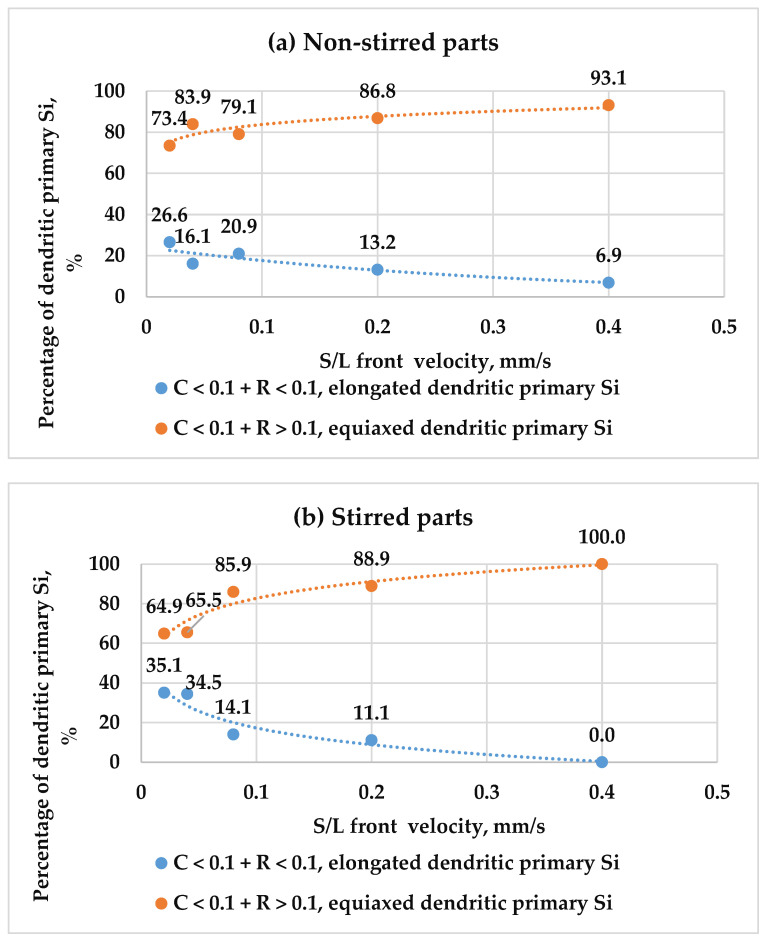
The percentages of elongated and equiaxed dendritic primary Si as a function of s/l front velocity in the (**a**) non-stirred and (**b**) stirred parts of the five samples.

**Figure 12:**
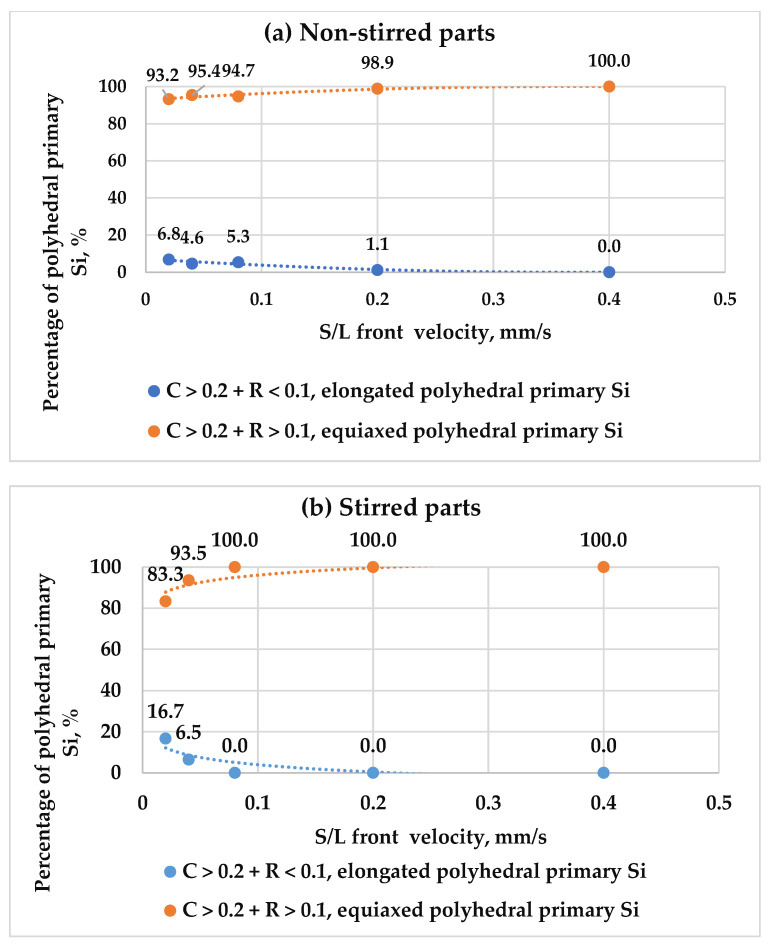
The percentages of elongated and equiaxed polyhedral primary Si as a function of s/l front velocity in the (**a**) non-stirred and (**b**) stirred parts of the five samples.

The values of roundness help to distinguish the elongated dendritic particles (C < 0.1 + R < 0.1) from the equiaxed dendritic particles (C < 0.1 + R > 0.1), and the elongated polyhedral particles (C > 0.2 + R < 0.1) from the equiaxed polyhedral particles (C > 0.2 + R > 0.1). This categorisation was applied to stirred and non-stirred parts across all samples (Table 2). Figure 11 shows how the percentage of elongated and equiaxed shapes in dendritic primary Si changes with s/l front velocity in both stirred and non-stirred sections. Similarly, Figure 12 displays these variations for polyhedral primary Si. An increase in s/l front velocity leads to a reduction in elongated dendritic primary Si and a rise in equiaxed dendritic primary Si. Similarly, a higher s/l front velocity decreases the percentage of elongated polyhedral primary Si and increases the percentage of equiaxed polyhedral primary Si in stirred and non-stirred parts. This can be attributed to the fact that increasing the s/l front velocity leads to an increase in the cooling rate, leading to a rise in the percentage of equiaxed particles and decreasing the percentage of elongated particles.

## 4. Conclusions

A conventional image analysis method was used to quantitatively characterise the primary Si particles in hypereutectic Al-Si alloys, focusing on their equivalent diameter and shape factors. The effect of forced melt flow by an RMF and s/l front velocity on the size and morphology of primary Si was investigated by solidifying samples with various front velocities at an RMF intensity of B = 7.2 mT and a temperature gradient of G = 8 K/mm. The main results can be summarised as follows:

- Increasing the front velocity during solidification resulted in an increased percentage of fine primary Si particles (<200 µm) and a decreased percentage of coarse primary Si particles (>200 µm), leading to the refinement of primary Si particles.
- Increasing the front velocity and applying an RMF together during solidification had a better effect on the refinement of primary Si particles.
- Increasing the s/l front velocity during solidification in non-stirred sections reduced dendritic percentages and increased star-like particles, while polyhedral percentages remained constant. The percentages of dendritic, star-like, and polyhedral Si shapes were relatively stable across different front velocities in stirred parts.
- Higher s/l front velocities decrease elongated dendritic and polyhedral primary Si particles, while increasing their equiaxed forms, in stirred and non-stirred parts.

## Figures and Tables

**Table 1:** The experimental parameters.

Name of Sample	$\mathrm{Solid}/\mathrm{Liquid}\;\mathrm{Front}\;\mathrm{Velocity},\;v_{SL}$ [mm/s]	Magnetic Induction of RMF, B [mT]	Temperature Gradient, G [K/mm]
A	0.02	0–7.2	8
B	0.04	0–7.2	8
C	0.08	0–7.2	8
D	0.20	0–7.2	8
E	0.40	0–7.2	8

**Table 2:** Dimensionless shape factors of various shapes of primary Si.

Shape Description	Shape Description	Average of Circularity	Standard Deviation	Average of Roundness	Standard Deviation
Polyhedral	Equiaxed polyhedral	0.59	0.07	0.58	0.16
	Elongated polyhedral (plate-like)	0.22	0.06	0.07	0.02
Dendritic	Equiaxed dendritic	0.08	0.01	0.33	0.07
	Elongated dendritic (feathery)	0.05	0.02	0.07	0.02
Star-like	Star-like	0.12	0.02	0.43	0.19

## Data Availability

The original contributions presented in the study are included in the article; further inquiries can be directed to the corresponding author.
